# Factors influencing reduced penetrance and variable expressivity in X-linked dystonia-parkinsonism

**DOI:** 10.1515/medgen-2022-2135

**Published:** 2022-08-12

**Authors:** Jelena Pozojevic, Björn-Hergen von Holt, Ana Westenberger

**Affiliations:** Institute of Neurogenetics, University of Lübeck and University Hospital Schleswig-Holstein, BMF, Building 67; Ratzeburger Allee 160, 23538 Lübeck, Germany; Institute of Human Genetics, University of Lübeck and University Hospital Schleswig-Holstein, Lübeck, Germany; Institute of Medical Biometry and Statistics, University of Lübeck and University Hospital Schleswig-Holstein, Lübeck, Germany

**Keywords:** X-linked dystonia-parkinsonism (XDP), retrotransposon insertion, repeat-length polymorphism, age-related penetrance, genetic modifiers

## Abstract

X-linked dystonia-parkinsonism (XDP) is a neurodegenerative movement disorder that primarily affects adult Filipino men. It is caused by a founder retrotransposon insertion in *TAF1* that contains a hexanucleotide repeat, the number of which differs among the patients and correlates with the age at disease onset (AAO) and other clinical parameters. A recent work has identified additional genetic modifiers of age-associated penetrance in XDP, bringing to light the DNA mismatch repair genes *MSH3* and *PMS2*. Despite X-linked recessive inheritance, a minor subset of patients are female, manifesting the disease via various mechanisms such as homozygosity, imbalanced X-chromosome inactivation, or aneuploidy. Here, we summarize and discuss clinical and genetic aspects of XDP, with a focus on variable disease expressivity as a consequence of subtle genetic differences within a seemingly homogenous population of patients.

## Introduction

X-linked dystonia-parkinsonism (XDP, DYT/PARK-*TAF1*, OMIM #314250) is a severe, neurodegenerative, and rapidly progressive movement disorder. The signs of XDP include dystonia and parkinsonism that generally start manifesting around the fourth decade of life [1], [2]. All XDP patients are of Filipino origin and descend from the common ancestor in whom the disease-causing variant initially arose. Given the position of this variant on the X-chromosome, XDP is inherited in an X-linked recessive manner and thus mostly affects men. The aim of our review is to summarize and discuss the factors contributing to the considerable variability in age-related penetrance and disease expressivity that has been observed in XDP patients, despite a seemingly identical genetic background. Furthermore, we will review the circumstances that account for the occasional presence of XDP signs in women.

**Figure 1:**
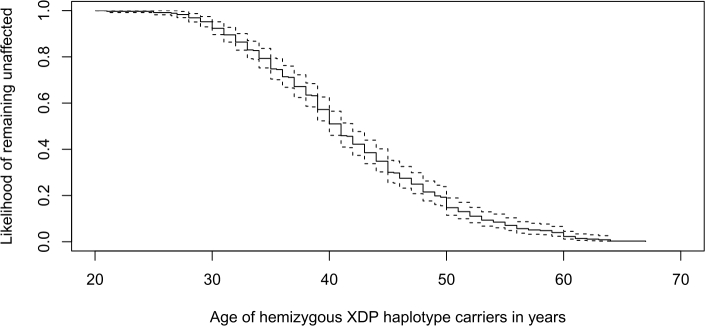
A Kaplan–Meier plot showing the increase in the age-related penetrance of XDP, i. e., a decrease in likelihood for men with the XDP haplotype to remain unaffected with increasing age.

## Clinical characteristics of X-linked dystonia-parkinsonism

In genetically tested patients, in whom the presence of disease-causing variants has been confirmed, the median age at onset (AAO) of XDP is 40 years [3]. However, the AAO spectrum is very broad (from 20 to 67 years) and penetrance increases with age (Figure 1 Kaplan Meier Survival Analysis) [3]. The majority (>80 %) of patients present initially with focal dystonia that usually generalizes within five years after onset (*dystonic phase*) [1], [2], [3]. Nevertheless, in 14 % of patients, parkinsonism signs are the first disease manifestation [3]. With time, dystonia and parkinsonism increasingly overlap (*combined dystonia-parkinsonism phase*), and in patients surviving this disease stage the predominant clinical picture is parkinsonism (*parkinsonian phase*) [1]. XDP patients have a shortened lifespan due to dystonia-related conditions (aspiration pneumonia or starvation) or suicide, and many of them do not survive longer than 10 years after the illness onset [1].

Of note, in patients that present with dystonia at onset, craniocervical dystonia is more commonly observed (∼60 %) than limb (∼37 %) or truncal (∼4 %) dystonia [4]. Approximately half of patients that initially exhibit craniocervical dystonia suffer from neck/shoulder dystonia, while 28 % and 23 % manifest blepharospasm and mouth/tongue dystonia, respectively [4]. Consistent with these findings, the initial report on 42 patients also found that first signs and symptoms occur less frequently in the legs than in other body parts (12 % head, 29 % axial musculature, 23 % upper extremities, 36 % lower extremities) [2]. Neuroanatomical changes in XDP affect the basal ganglia and cerebellum, with striking striatal atrophy and iron deposition that correlates with the degree of atrophy, disease duration, and disease severity [5], [6], [7].

## Genetic basis and molecular mechanisms of XDP

XDP is endemic to the Philippines and it is particularly prevalent on Panay Island (5.74 in 100,000 individuals) [1], where a genetic founder likely originated. Indeed, all XDP patients inherited from this founder a common haplotype that consists of seven changes located within or surrounding the *TAF1* gene on the X chromosome. This XDP haplotype encompasses five disease-specific single-nucleotide changes (DSCs), a 48-bp deletion, and an SVA (SINE-VNTR-Alu) retrotransposon insertion [8], [9]. Importantly, none of these changes are situated in the protein-coding regions or present in >470 ethnically matched controls [10], leaving the identity of the actual XDP-causing variant unclear. The first experimental evidence indicating the culprit emerged from studies that edited the XDP-relevant SVA retrotransposon out of patient-derived cells. This approach led to a successful rescue of molecular phenotypes, such as the decrease in *TAF1* expression and the partial retention of the intron containing the SVA insertion, in the edited cells [11], [12], [13].

Namely, the XDP-relevant SVA retrotransposon is inserted in intron 32 of *TAF1*, and early research showed that a neuron-specific transcript of *TAF1* was reduced in the caudate nucleus (dorsal striatum) of an XDP patient in comparison to controls without the XDP haplotype [9]. *TAF1* encodes the largest subunit of the general transcription factor TFIID that is a part of the preinitiation complex, which is indispensable for proper promoter recognition and transcription by RNA polymerase II (reviewed in [14]). Subsequent studies demonstrated a consistent downregulation of *TAF1* transcripts in various tissues and cell lines, with the *TAF1* being the only gene in the disease-linked region that shows changes in expression in XDP patients [11], [12], [15]. This effect on transcription is not surprising and has been well-documented for SVA retrotransposons in the human genome. More than 2,700 SVA elements have been found in the human genome reference sequence [16]. Although they are typically heavily methylated and thus silenced, these elements may regulate gene expression depending on the genomic context, i. e., location of their insertion [17]. Furthermore, the XDP-relevant SVA retrotransposon was shown to cause the production of an aberrant *TAF1* transcript that contains a deep intronic part of intron 32 proximal to the insertion and terminates 716 bp 5´ of the SVA [12]. The reduction in the *TAF1* expression and the retention of a part of intron 32 in some *TAF1* transcripts are currently considered relevant cellular/molecular XDP phenotypes, but their role in XDP pathogenesis is not defined and warrants further investigation.

**Figure 2:**
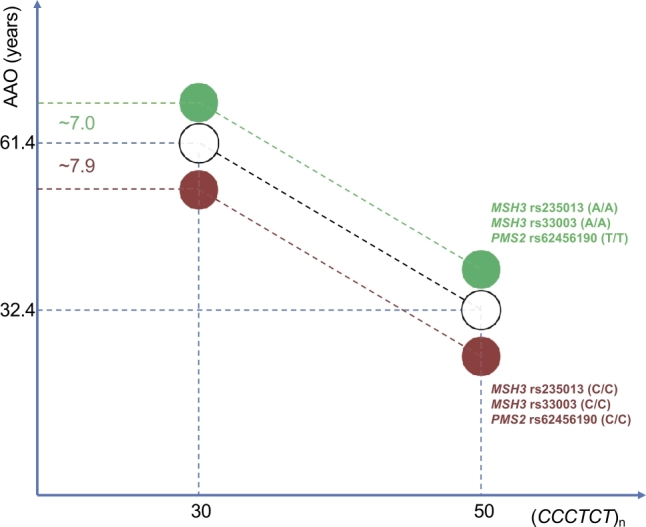
A diagram showing the effect of the number of (*CCCTCT*)_n_ repeats, alone, on age at onset (AAO) of XDP patients (white circles) and the combined effect of the harmful alleles and the number of repeats (red circle) and protective alleles and number of repeats (green circle). The latter two decrease the AAO by 7 years and increase the AAO by 7.9 years, respectively.

## Genetic modifiers of XDP and their potential modes of action

### (CCCTCT)_n_ repeat within the SVA retrotransposon

The pathogenic role of the SVA retrotransposon insertion was further substantiated by the discovery that it contains a polymorphic hexanucleotide repeat, the length of which inversely correlates with AAO in XDP patients (Figure 2) [18]. Canonical SVAs contain five consecutive regions: a polymorphic hexamer (*CCCTCT*)_n_ repeat at the 5’ end, followed by an Alu-like, a VNTR (variable number of tandem repeats), a SINE (short interspersed nuclear element), and a poly(A) domain at the 3’ end. While the average SVA length is reported to be ∼2 kb, their size can vary due to changes in copy number in their repetitive domains [19], and the XDP-specific SVA is ∼2.6 kb long. In thus far investigated XDP patients, the polymorphic (*CCCTCT*)_n_ repeat number ranges between 30 and 55 repeats [4]. This variability explains approximately 50 % of the variance in XDP AAO [4], [18]. Interestingly, patients presenting with parkinsonism at onset have a smaller median repeat number and later median AAO in comparison to those showing dystonia as the initial manifestation (39 vs. 41, $p=1.8\times 10^{-3}$ and 46 vs. 40, $p=8.0\times 10^{-5}$). This relation between the repeat number and dystonia/parkinsonism at onset is indirect and determined by the AAO [4]. That is, the number of repeats influences AAO which further affects the signs at onset, but whether a patient will initially become affected by dystonia or parkinsonism is not defined by the variability in the repeat number. In patients that present with dystonia at onset, the repeat number does not seem to be correlated with whether the disease will start in the craniocervical region, arm, leg, or trunk [4]. However, within the craniocervical region, the patients whose mouth/tongue first becomes affected have a slightly lower repeat number than those initially suffering from blepharospasm [4]. Furthermore, we described a positive correlation of the (*CCCTCT*)_n_ repeat number with the severity of dystonia (i. e., the higher Burke-Fahn-Marsden Dystonia Rating Scale score) while a positive correlation with the severity of parkinsonism and cognitive dysfunction was indicated but did not reach statistical significance likely due to the low number of investigated patients [4].

The mode of action through which the hexanucleotide repeat modifies the age-related penetrance and other clinical characteristics of XDP is currently unknown. In a subset of patients, blood-derived *TAF1* mRNA levels were negatively correlated with the number of repeats, indicating that the repeat number may influence the previously observed reduction of *TAF1* expression. Additionally, and similarly to what was described for other repeat-expansion disorders (e. g., myotonic dystrophy (DM), fragile X tremor ataxia syndrome (FXTAS), C9orf72-associated amyotrophic lateral sclerosis and frontotemporal dementia (ALS/FTD)), if the XDP-relevant hexanucleotide repeat is transcribed, scenarios such as RNA foci formation [20] and repeat-associated non-ATG (RAN) translation [21] may be involved not only in modifying, but even in causing this disease. RNA foci are accumulations of RNA fragments comprised of repeated nucleotides that adopt unusual secondary structures, sequester various RNA-binding proteins, and as such become toxic to the cells. Alternatively, or in parallel, neurodegeneration may result from toxic species such as RAN-translated dipeptides.

### DNA mismatch repair and repeat instability

Recently, a genome-wide association study (GWAS) was performed to uncover additional genetic factors that influence the age-related penetrance of XDP. The study found three independent genomic loci that account for an additional 25.6 % of the remaining variability, not explained by the repeats, and 13.0 % of the overall variance in AAO in XDP [22]. Two of these loci are located within or immediately adjacent to the *MSH3* gene on chromosome 5, while the third locus includes signals on chromosome 7, adjacent to the *PMS2* gene [22]. Importantly, the combined effect of the protective alleles of the lead SNPs in each of the three regions delays XDP onset by seven years (Figure 2). The *MSH3* and *PMS2* genes code for proteins that are components of the DNA mismatch repair (MMR) cellular machinery, which is in charge of recognizing and repairing mismatched base pairs in DNA. The MMR response to larger insertion/deletion loops (up to 13 nucleotides) in particular [23] is initiated by recognition of mismatched DNA by the MutSβ complex, comprised of MSH2 and MSH3. This is followed by the recruitment of MutLα (MLH1-PMS2) heterodimers, which introduce single-strand breaks near the mismatch and enable exonucleases to degrade the mismatch-containing region. Remarkably, one of the loci in *MSH3* modifying AAO in XDP has been associated, also by GWAS, with disease progression in Huntington’s disease (HD) [24], and signals in *MSH3*, *PMS2*, and other DNA maintenance genes modify AAO in HD and DM [25], [26]. The proposed mechanism of disease modification of the MMR machinery is that it recognizes longer stretches of repeats that are partially unwound and, in an effort to repair them, instigates instability, i. e., increases the length of the repeat tract. Indeed, the XDP-relevant hexanucleotide repeat displays instability in terms of intergenerational transmission and somatic mosaicism. Namely, it has been reported that in XDP families, repeat numbers tend to increase in successive generations and are more likely to be larger than in the previous generation if they are inherited from a mother than from a father [4], [18]. Furthermore, the hexanucleotide repeat exhibits somatic mosaicism in XDP patients, reflected in a higher repeat number in brain tissues compared to blood, suggesting a tissue-specific and potentially even brain-region specific effect [4], [27].

Although the exact modifiers of AAO in XDP patients, tagged by the three lead signals on chromosomes 5 and 7 are currently unknown, it is conceivable that they act by regulating expression, function, or even location of the MSH3 and PMS2 proteins. Interestingly, exon 1 of *MSH3* contains length polymorphisms, the length of which inversely correlates with *MSH3* expression and AAO in XDP, HD, and DM patients [22], [26]. The same polymorphism may have a role in regulating the levels of MSH3 in the nucleus. Specifically, nuclear localization (NLS) and nuclear export (NES) signals have been identified within MSH3 as responsible for the ability of this protein to cross the nuclear envelope [28], [29]. Furthermore, deacetylation of MSH3 by histone deacetylase 3 (HDAC3) increases repeat expansions/instability [29]. The deacetylation sites in MSH3 overlap with NLS1, and MSH3 localization is partially dependent on HDAC3 activity. Importantly, NLS1 (encoded by *MSH3* exon 2) is in close proximity to the length polymorphism in exon 1, and it has been shown that the shortest MSH3 allele is more prone to staying in the cytoplasm in comparison to the wild type protein [28]. This is in agreement with the increase in AAO that was observed in patients carrying the shortest allele, as the absence of MSH3 from the nucleus would prevent it from introducing instability and would thus be protective. Whether this length polymorphism in exon 1 of *MSH3* is the genuine modifier behind one of the three signals seen in the XDP GWAS and what the other two are, remains to be addressed in the future studies.

**Figure 3:**
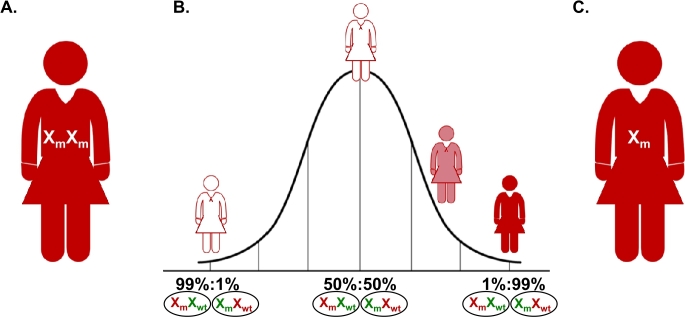
Explanations for penetrance of XDP in women. A. A homozygous carrier of the XDP haplotype. B. A Bell curve showing the effect of X-chromosome inactivation (XCI) on the penetrance of XDP in heterozygous female XDP-haplotype carriers. Xm – affected X chromosome (i. e., X chromosome containing the XDPhaplotype), Xwt wildtype X chromosomes. The silenced X chromosome is shown in red and the one that is expressed in green. The percentages below the curve are the ratios of cells with silenced affected vs. unaffected X chromosome. White female figures – unaffected heterozygous carriers; light and dark red female figures, mildly or severely affected female XDP patients, respectively. C. A woman with X chromosome monosomy (45, X), harboring only the affected X chromosome in all her cells.

## Penetrance of XDP in women

In the majority of female carriers of the XDP haplotype, X chromosome inactivation (XCI) is balanced, i. e., a sufficient number of cells express solely the wildtype X chromosome for these women to remain healthy, which is a defining characteristic of the X-linked recessive inheritance. Nevertheless, occasionally female carriers of the XDP haplotype manifesting XDP are encountered in clinical practice. To date, 16 such women have been reported in the literature, and in comparison to men, they seem to have a milder phenotype, later AAO, and a milder course of the disease [30], [31], [32], [33]. There are three possible explanations for the occurrence of XDP symptoms in females. Firstly, they may be homozygous carriers of the XDP haplotype (Figure 3A) and two such women have been hitherto described [30], [33]. Secondly, the affected women may have an imbalanced XCI, “skewed” towards the preferential expression of the affected allele. Namely, although in most women XCI ranges between 80 %:20 % and 50 %:50 % ratios of cells expressing one X chromosome vs. the other, a small percentage of females have a skewed ratio (>80 %:20 %), and thus a bias towards cells expressing one or the other X-chromosome. Interestingly, skewing increases with age, and the percentage of individuals with a >80 %:20 % XCI ratio was found to be almost three times higher among adult women (14.2 %) compared to newborn girls (4.9 %) [34]. The women whose XCI is skewed towards the preferential expression of the X chromosome that contains the SVA insertion will thus have a higher likelihood of becoming affected. Indeed, this skewing that occasionally can be extreme, accounting for less than 5 % of cells in the patient’s body expressing the wildtype allele [31], is the most frequent explanation for the penetrance of XDP in women (Figure 3B). The third and the least common scenario leading to XDP expression in a female is a typical or atypical Turner syndrome, i. e., complete (45,X) or mosaic (45,X/46,XX) X chromosome monosomy, resulting in a loss of the wildtype X chromosome in all or some cells of the affected woman (Figure 3C). Under these circumstances, the XDP-relevant genetic setting is comparable between a typical male XDP patient and a Turner syndrome XDP patient. Of note, atypical Turner syndrome, in particular, may go undiagnosed because of the mild, unremarkable phenotype is appreciated only upon XDP-relevant genetic testing [32]. Interestingly, in terms of X-chromosome aneuploidies, Klinefelter syndrome, a relatively frequent (1:500 to 1:1,000 males) condition characterized by a presence of an additional X chromosome in a male genotype (47,XXY), may be protective and reduce XDP penetrance in individuals heterozygous for the XDP haplotype with balanced XCI.

## Concluding remarks

Even though all XDP patients can track their ancestry to the Panay island in the Philippines and share the common haplotype, there are subtle differences among this seemingly genetically homogenous population. In addition to the polymorphic hexanucleotide repeat within the SVA, modifiers in MMR genes determine age-related disease penetrance. Combined, these genetic factors explain approximately 63 % of AAO variability in XDP patients, indicating that additional modifiers remain to be identified. Understanding the molecular mechanisms of disease modification has a multifaceted benefit for XDP patients that includes improved genetic counseling, more efficient selection and enrollment of patients in clinical studies and trials, and may even lead to the development of specific therapies. While this important newly acquired knowledge is applicable to all the mutation carriers, additional factors that might modify disease expressivity in isolated cases should be considered. This is illustrated by the rare female patients reported to manifest XDP due to epigenetic and copy-number variation, including imbalanced XCI and X-chromosome monosomy.
